# On the Automobile1From “A Few Remarks,” by Simeon Ford. Copyright, 1903, by Doubleday, Page & Co.

**Published:** 1904-06

**Authors:** Simeon Ford

**Affiliations:** New York


### On the Automobile.[1]

By SIMEON FORD, New York.

OUR streets have always been hard enough to navigate, heaven knows, but nowadays, with the electric trolleys and the automobiles added, pedestrianism has degenerated into a mere succession of frenzied leaps and convulsive stops, and our progress to and fro is like that of the startled fawn, which

> "Bounds from crag to crag,
> Hearing the hunter's horn."

Shakespeare, who was up to date and a little ahead of it, said:

> "No man means evil but the devil, and we shall know him by his horns."

1. From "A Few Remarks," by Simeon Ford. Copyright, 1903, by Doubleday, Page & Co.

This eternal horn-blowing is a nuisance and a nerve-destroying crime, and is unnecessary and silly. I have noticed that the smaller the auto the bigger the horn. To hear one of these little tin washboilers, with a one-horse-power engine and a twelve-horse-power horn and a twenty-mule-power driver, coming down the avenue, you'd suppose Gabriel with his trump had broken loose at last, and when you look up, expecting to see a trump, you see nothing but a two-spot.

I don't claim that every man who runs an auto is a jackass, but I do claim that every jackass runs an auto. I run one myself.

But when I run over a pedestrian, I just mow him down in a quiet, dignified and refined manner, and don't add insult to injury by frightening him to death before I kill him.

I am an automobilist, not from choice, but in self-defense. Some achieve automobiles and some have automobiles thrust upon them. I live in a suburban town which was early seized with automobiliousness in its most virulent form. One of my immediate neighbors bought a machine of limited capacity for everything but noise. Its capacity for noise was unlimited. It was also long on smell. At first the rest of us talked of tar and feathers. Some of us thought that was too mild and that the punishment should fit the crime. And while we hesitated, we all got the craze, and now we are all tarred with the same stick.

This was my daily program for a time: I would start to drive to the station. Presently the earth would tremble, my horses would tremble, my coachman would tremble, and I would tremble most of all, and with rumblings and snortings and smells indescribable, my neighbor would dash by. I would then breathe a prayer, disentangle my horses from a barbed-wire fence, pluck my wagon from a nearby tree, reconnect them, and proceed on my way rejoicing. Presently I would overhaul my friend. He and his chauffeur would be reclining on their backs under the auto, doing stunts with spanners and monkey wrenches. I would then take my neighbor into my wagon, drive him to the station, and his machine would wait to be towed home by my team.

My neighbor argued that the auto was the coming mode of locomotion, and that the horse must go. I agreed with the latter proposition. I reminded him that one of my best horses, hearing his approach, decided that he must go, and that I thought he was going yet. I stayed with him awhile, but decided he was too swift a proposition for me to keep company with. I never could decide whether it is the appearance of the machine, or the smell, or the raiment of the driver that gets into a horse's nerves, but I reckon it's the raiment. The spiritual description of the Lily of the Field applies to them pretty well: "They toil not, neither do they spin, but verily I say unto you that Solomon in all his glory was not arrayed like one of these."

The first machine I looked at was small, simple and inexpensive. It had but one cylinder. The salesman said that was an advantage. He said a four-cylinder engine would get out of order four times as often. This machine had a handle on the side like a barrel-organ. He showed me how to make it go fast, and slow, and stop, and start, and all while the machine stood in the store. A child of ten years could run it, he said. "Now, if you want to get out of a tight place," he said, "get a sudden move on—you touch this lever called the accelerator."

He touched it, and with that something went wrong, and the handle I have alluded to flew around and smote him violently in the abdomen. When he came to I told him a child of ten might run the machine, but the child would have to have a very strong stomach.

Next, a friend took me out in a base-burning steam vehicle. He had a third man with him, and I sat behind on a sort of broiler arranged over the boiler. The day was warm, and I understood at once why the machine was called a "steamer." I felt like the nigger who used to squat on the safety-valve on the Mississippi boats. I amused myself by watching the steam-gage and wondering how long it would take me to come down if anything went wrong. The exhaust steam went up my trouser's leg and I felt like the squid, which scientists say envelops itself in a cloud or fog of its own making in order to conceal itself from its enemies. My friend, meanwhile, explained the mechanism, but I told him if I had to become a master mechanic it would pay me better to go and run the *Kaiser Wilhelm*.

The consensus of opinion seemed to be that the gasoline machine was the thing. There was power, simple and direct! It ran by a series of explosions. That appealed to me at once. That's the way my hotel on Park avenue has been run during the past year—by a series of explosions of dynamite by the tunnel people, and a series of explosions of profanity on the part of myself and my few remaining boarders.

Every auto I thought of buying all my friends assured me was no good, and in the light of subsequent experiences I guess they were right. Finally, on my own responsibility, I bought that lovely lobster-pink creation in which I may be seen 'most any pleasant day now running merrily through the park or street and anon sitting reposefully while my chauffeur, assisted by the populace, explores the vitals of the machine, looking for trouble. I remember when I was a boy I saw and admired at Barnum's

Museum a working model of an engine all made of glass, but I never dreamed I should own one.

I am getting proud of my machine. I think it holds the record for having traveled fewer miles in a given time than any other yet devised. My engine will break when standing motionless on the barn floor, simply through the power of gravitation. It is operated by a skilled mechanic and costs me as much per month as it would to run the *Corsair*. But it has one merit. I never wander so far from my own fireside but that I can easily walk back. I have worn out six sets of hinges in the hood peering at the engine to see what is busted.

I used to get up and help the chauffeur to look, until one day when we were both hidden behind the hood a sneak carried off my fur robes. Now I just sit back and listen to the jeers of the populace and sigh to think of the happy times gone by when I used to travel on the street cars and get to my destination on the same day.

